# Health-Related Quality of Life and Outcome after Total Knee Replacement: Results from a Cross-Sectional Survey in Jordan

**DOI:** 10.1155/2021/5506809

**Published:** 2021-12-10

**Authors:** Yazan Al Thaher, Othman A. Alfuqaha, Ahmed Dweidari

**Affiliations:** ^1^Faculty of Pharmacy, Philadelphia University, Amman 19392, Jordan; ^2^Scientific Research Office, Jordan University Hospital, The University of Jordan, Amman 11942, Jordan; ^3^Department of Special Procedure, Division of Orthopedics, School of Medicine, The University of Jordan, Amman 11942, Jordan

## Abstract

Total knee replacement (TKR) is a common procedure that is considered cost-effective with excellent long-term survivorship. This cross-sectional study, which includes 118 selected patients in Jordan from January 2020 to January 2021, aims to assess health-related quality of life (HQOL), functional status, and associated factors before and after 3 to 12 months of TKR. Furthermore, several demographical factors were included in this study to evaluate the potential association with the procedure's outcome. Western Ontario and McMaster Universities Osteoarthritis Index scale (WOMAC) and Short Form-36 (SF-36) scale were used in this study. It was found that the main change occurred 6–12 months after the procedure. Gender and age were found to be the primary predictor of quality of life among TKR patients. Patients before the TKR procedure experienced severe pain, joint stiffness, physical impairment, and psychological demand issues. After the TKR procedure, all symptoms were improved significantly. The TKR procedure can improve the quality of life for patients, which can be detected for several months after the procedure. Function and pain are the most critical indicators of improvement in quality of life. Assessing the quality of life is a key indicator for identifying patients without improvement, as well as improving the health care process and comparing other alternative interventions.

## 1. Introduction

Osteoarthritis of the knee is a prevalent problem that causes pain and functional limitations, especially in the elderly population (>80% in population above 65 years) [1]. Common treatments for osteoarthritis involve the use of medications (e.g., nonsteroidal anti-inflammatory drugs) [2]. Other treatment approaches involve physical therapy, for example, backward walking on static stability [3], topical sesame oil [4], and topical *Linum usitatissimum* L. (flaxseed) oil [5]. End-stage osteoarthritis, disability, and loss of HQOL all necessitate TKR, which relieves pain, improves long-term function, and restores mobility. The TKR is one of the common procedures performed globally and is considered cost-effective with excellent long-term survivorship, with substantial variations in rates across different countries [6, 7].

Although most patients have a favorable clinical outcome after TKR, several studies have reported that more than 20% of patients are dissatisfied with the outcome after procedure [8–10]. This deficiency in patient satisfaction drives research and development in this field. Improving outcomes can be done by changing implant design, implantation methods, improving preoperative care for patients, etc. Therefore, measuring patients' quality of life and their perception of the procedure is of paramount value to improve this procedure and increase the proportion of patients who have successful outcomes and improved quality of life [10].

A highly effective evaluation for the outcome after TKR is essential for making patient-reported outcome measures. The outcome evaluation should account for different aspects of the patient's life, including activity and functional aspects, social aspects, as well as psychological or emotional aspects [9]. Several scores are developed for this purpose, which features the patient in the assessment of the TKR outcome. These scores can be divided into two types: those measuring HQOL for aspects that can be influenced by feelings of physical well-being or mental state and those evaluating the knee function and pain. Examples of the former are the 36-Item Short Form (SF-36) and 12-Item Short Form (SF-12) that are not specific for the outcome after TKR. However, more specific scores are available, such as the Osteo Arthritis Knee and Hip Quality of Life (OAKHQOL) and the Knee Quality of Life-26 (KQOL-26). The second type of score is called functional scores, which evaluate the knee function and pain, such as the Knee Society Score, WOMAC, and Oxford Knee Score. A combination of these two types of scores is mostly used to evaluate the outcome after TKR [11, 12].

Most studies in the literature use one generic score from the first type previously mentioned and one specific score from the second type [9, 13]. The two questionnaires complement each other and measure different aspects of patient outcomes. The SF-36 and WOMAC scores are the most commonly used combination in literature [14]. While the WOMAC score measures the joint's stiffness, pain, and physical functioning, the SF-36 measures HQOL for the patient.

This study aims to assess HQOL, functional status, and associated factors before and after TKR, consequently, measuring the clinical effectiveness or outcome of the TKR. There was no recent multicenter data regarding preoperative or postoperative measurement for HQOL or patient outcomes in Jordan. This helps identify patient satisfaction deficiencies to make future patient-informed measures to this procedure and improve current clinical practice. The results will be important for orthopedics and physicians who treat TKR patients. Herein, HQOL was evaluated before and after TKR. Moreover, patients' HQOL was evaluated in patients after 3 to 12 months after TKR. Several demographical factors were included in this study to assess any potential association with the procedure's outcome.

## 2. Materials and Methods

### 2.1. Design

This cross-sectional study evaluates the HQOL, outcome, and functional status before and after TKR among patients in Jordan. The authors adhered to STROBE guidelines.

### 2.2. Settings and Participants

The population of this study comprises all patients on the waiting list for TKR who have been diagnosed with knee osteoarthritis and patients who have had TKR. This study was conducted in a tertiary hospital in Amman (the capital of Jordan) since they had the largest number of TKR patients in Jordan, among other Jordanian cities. All patients admitted to the hospital for TKR in 2020 were invited to participate in this study. According to a previous study [15], a total of 100–300 samples will have a confidence interval of 95% and an error estimation of 5%. Exclusion criteria were patients with a road traffic accident that caused TKR, malignant tumors, and psychiatric disorders. Consecutive patients with primary TKR in the selected hospital were eligible to participate in this study. In the selected hospital, a total of 200 TKR patients had the procedure in 2020 in the selected hospital. A convenient sampling process was selected to collect the data over one year (from January 2020 to January 2021). A total of 118 patients were followed up and completed an envelope survey, with a response rate of 59%. All patients received a letter informing them about the study and asking for voluntary participation. Patients were followed before TKR and up to a year after procedure.

Patients visited the orthopedic clinic within the first 3 months and 3 to 6 months after having a TKR procedure. During their visit, data were collected through an interview. Patients were contacted 6 months to 12 months after TKR procedure by phone for data collection because they usually do not come to the clinic unless serious complications happen. However, all participants completed the SF-36 and WOMAC scores, as well as additional questions about the clinical disease aspects and sociodemographic information. The data were collected from participants before and after the procedure within one year after the procedure. After the procedure, patients who did not come to the clinic were contacted via phone call. The ethical guidelines of scientific research were followed during the interview and phone call.

### 2.3. Data Measurement

Patients were asked to complete the following scales.

#### 2.3.1. Part One

Sociodemographic characteristics of patients included gender, age, marital status, educational level, weight, height, body mass index (BMI), location, site of knee procedure, previous TKR procedure, and comorbidities.

#### 2.3.2. Part Two

WOMAC scale was used in this study. This scale consisted of 24 items divided into three dimensions: pain severity (5 items), joint stiffness (2 items), and daily physical daily functioning of the joint (17 items). A five-point Likert type scale was used to answer the items of WOMAC scale from 1 “None” to 5 “Extremely.” It is widely recognized that the WOMAC scale has reliable psychometric properties [16]. Alfatafta [17] recently found that the Arabic WOMAC scale is an effective tool to evaluate the quality of life among Jordanian patients after a TKR. Therefore, the Arabic version of the WOMAC scale was used, and its validity and reliability were checked. The five-point Likert mean score has a mild mean score from 1 to 2.33, a moderate mean score from 2.34 to 3.67, and a high mean score from 3.68 to 5. A high mean score represents severe pain, stiffness, and difficulties in performing daily activities.

#### 2.3.3. Part Three

SF-36 was used in this study. It consists of 36 items divided into two main dimensions: physical demand (22 items) and psychological demand HQOL (14 items). Since the WOMAC scale measures the physical demand, the psychological demand section was only taken from SF-36 to measure different aspects of psychological issues such as emotional problems, depression, worn out, downheartedness, and lack of accomplishment. The Arabic version of SF-36 was used after obtaining the necessary permissions [18], and this scale's validity and reliability were checked. A five-point Likert type scale was used to answer the 14 items of the SF-36 scale from 1 “None” to 5 “Extremely.” The cut-off point for this scale was measured the same as for the WOMAC scale. A high mean score represents severe emotional problems, downheartedness, blue, and lack of accomplishment.

### 2.4. Validity and Reliability

Validity and reliability were assessed for the Arabic scales used in this study. Cronbach's alpha, correlation coefficient, Kaiser–Meyer–Olkin (KMO) test, and Bartlett's test of sphericity were calculated (presented in Table 1).

As shown in Table 1, Cronbach's alpha for the WOMAC scale and SF-36 were considered applicable. Pearson correlation coefficient ranged between 0.31 and 0.71 for the Arabic WOMAC scale, and its dimension indicated a discriminant validity due to moderate correlation. A positive correlation coefficient was found between the total WOMAC scale and (SF-36) psychological demand. The KMO test was 0.75 for the Arabic WOMAC scale, which is considered adequate (>0.70) [19]. Bartlett's test of sphericity *p* < 0.05 was deemed significant [19]. Regarding (SF-36) psychological demand dimension, the KMO test was 0.76 and Bartlett's test of sphericity was significant. These results suggested that the Arabic WOMAC scale and Arabic SF-36 were valid and reliable for this study.

### 2.5. Statistical Analysis

The Statistical Package for the Social Sciences (SPSS) version 22 was used to analyze the study results. Descriptive statistics, multiple linear regression analysis (stepwise method), Pearson correlation coefficients, and *t*-test were used in this study.

### 2.6. Ethical Considerations

Ethical approval was obtained from the institutional review boards in the selected hospital (No. 10/2020/21599) before collecting the information for this study. The study's primary aim was explained to all patients, and they were informed about their right to refuse or withdraw from the study. Moreover, patients were informed about confidentiality and anonymity during data analysis.

## 3. Results

One hundred eighteen patients participated in this study, with a total response rate of 59% of the total number of participants (200 patients). Females represented 53.8% of the sample. All participants were married and had a mean age of 66 years, and the majority lived at central of Jordan (Table 2).

The educational level for most of the participants was high school or less, while participants' average BMI was found to be overweight (26.17 ± 3.32). The location of the knee procedure was almost equal between the right and left sides. Diabetes mellitus and hypertension were found to be the most common comorbidities among participants in this study.

The differences in quality of life among participants are shown in Table 3, where the quality of life was calculated before and after TKR and means, standard deviations, percentages, *t*-test, and *p* value.

Before the TKR procedure, patients experienced severe pain, joint stiffness, physical impairment, and psychological demand issues. Physical functioning impairment before TKR procedure exhibited the highest mean score on the WOMAC scale. On the other hand, psychological demand (SF-36) in patients before TKR reported severe anxiety levels, downheartedness, emotional problems, and lack of accomplishment. After TKR procedure, there was a significant improvement in all dimensions of the WOMAC scale, with an overall mild level. Physical functioning impairment was decreased significantly after the TKR procedure. Psychological demand in SF-36 decreased significantly to a moderate level. Furthermore, the quality of life after TKR procedure was improved significantly, as expressed by the total WOMAC scale.

During the data collection period, participants were added into 3 major groups according to the date of follow-up after a TKR procedure. A total of 38 participants were seen in orthopedic clinics within the first 3 months of the TKR procedure (group 1), 48 participants were seen between 3 and 6 months after the TKR (group 2), and a total of 32 participants were contacted by phone from 6 months to 12 months after the TKR (group 3). The demographic factors of these three groups are shown in Table 4.

Moreover, means, standard deviations, and overall level were calculated to measure the quality of life among these groups, as shown in Table 5.

Regarding the psychological demand, a *t*-test was performed between groups 1, 2, and 3 and the results showed no significant association between all three groups (*p* > 0.05). Regarding the total quality of life, there was a significant difference between groups 1 and 2 (*t* = 4.99, *p* < 0.001) and between groups 1 and 3 (*t* = , *p* < 0.001). However, there was no significant difference between groups 2 and 3 (*t* = 0.23, *p* < 0.82). Group 3 had the lowest mean score among other groups, with an improved quality of life status. In the current study, the best improvement in the quality of life was from 6 to 12 months after TKR. Overall, patients presented lower levels of pain, stiffness, and psychological issues.

Finally, linear regression analysis (stepwise method) was conducted (as shown in Table 6) to study the contribution of many demographic factors such as gender, age, marital status, educational level, BMI, location, qualification, and site of the knee procedure in predicting quality of life outcome.

Gender and age are the main predictors of quality of life among TKR patients, with a total variation of 39.4%. While gender was found to account for 37.1% of the variances in quality of life (*R*^2^ = 0.371, *p* < 0.001), age was a significant predictor of quality of life with a total variation of 2.3% (*R*^2^ = 0.023, *p* < 0.05). Descriptive statistics were performed to determine which gender type and age categories had more levels of quality of life after TKR procedure. The results showed that male patients had higher levels of quality of life than female patients (mean_male_ = 2.06, mean_female_ = 1.80). Based on age categories, mean ages below 65 years are associated with a higher quality of life after TKR. However, other factors such as marital status, educational level, BMI, location, qualification, and site of knee procedure were excluded as predictors in this study.

## 4. Discussion

The results of this study provided information about the quality of life and outcome of TKR patients. This study provides information about pain, stiffness, physical impairment, and psychological demand issues before TKR and 12 months after the intervention. The main change was found to occur 3–6 months after the procedure. The WOMAC progress score decreased from severe to mild before and after the TKR procedure. Moreover, the psychological demand (SF-36) scale was changed from severe to moderate after the TKR procedure. Following the intervention, patients' physical and psychological assessments changed significantly.

Elderly patients had longer hospital stays and higher mortality and morbidity compared to younger patients. However, no significant differences in functional performance and pain were found between elderly (>65 years) and younger patients (<65 years) [20]. In another study, elderly patients' differences in everyday comfort and pain-related scores were significant between preoperative and postoperative evaluations [21]. In a study including 661 young patients with an average age of 54 years, it was found that 33% had swelling, 33% had residual pain, and 41% had stiffness after TKR [22]. In contrast, another study highlighted that advanced age and complications such as pulmonary disease limited the gains in scoring and reaching a satisfactory quality of life [23]. In this study, younger patients had a better quality of life compared to elderly patients. It was found that the lower the age of TKR patients, the more improved the quality of life or procedure outcome.

There is evidence that men's activity levels and functional scores after TKR are higher than in women [24]. The negative result of the female gender, especially on stiffness and residual pain after TKR, has been established by others [9]. Moreover, females with low social support and primary educational level, and patients with worse mental health before procedure, presented a poorer HQOL after TKR [13]. Our results agree with previous studies, where male patients are associated with better outcomes after TKR compared to female patients.

Obesity had been associated with worse scores in all dimensions of the WOMAC score. It negatively affected the initial and long-term assessment results and predicted poorer patients' quality of life [12]. Dietary and lifestyle modifications aiming at obesity improve patients' overall quality of life. In addition, TKR had a positive outcome on BMI and functional outcomes over the long term, with weight loss observed in 31% of cases obviously because of retrieving mobility after procedure [25]. In this study, no association was found between obesity and quality of life in the study participants.

Patients with comorbidities such as arthritis, lupus, and multijoint disease have the most significant functional improvement, especially in most severe cases [9]. However, it has been reported that WOMAC and SF-36 predictors of function and pain scores are increased with increasing comorbidities and low preoperative SF-36 mental health scores [26]. Patients with poor psychological status, for example, anxiety and depression, had a high dissatisfaction rate despite having functional improvements after TKR [9]. Other demographic factors, such as educational level and housing location, had no effect on the outcome after TKR, and they were not good predictors of quality of life after TKR [26]. In our study, no association was found between comorbidities and quality of life.

Improvement in functional aspects after the initial assessment was reported in several studies. Kilic et al. [27] used the SF-36 and Knee Society Clinical Rating System (KSCRS) to evaluate patients after 6 weeks and 6 months; significant improvements were observed in all scales after 6 months. Nevertheless, the physical dimension continued to improve significantly till 6 months. Bruyère et al. [28] maintained the observed improvements in emotional and functional state dimensions in WOMAC and SF-36 until the follow-up ended after 7 years.

Fernandes et al. [29] observed excellent improvement in 28 patients who performed TKR after 6 months. The balance and quality of life were assessed using WOMAC, Short Form Health Survey, and McMaster Universities Osteoarthritis Index questionnaire. However, despite the improvement, the level of physical activity did not reach the level expected for healthy individuals. Furthermore, the habit of practicing physical activity seems to influence the level of activity more than the treatment itself.

Many studies have found that pain levels improve in the first few weeks and even several years after procedure [26]. The improvement of pain is strongly correlated with improving the scores in different scales and the patient's quality of life after the procedure. Therefore, persistent pain in the assessments after procedure is negatively correlated with the possibility of attaining a good outcome. Moreover, pain and joint function improvements are closely associated with physical activity and social support before the procedure [30].

It is essential to mention some limitations of this cross-sectional study. The SF-36 and WOMAC scores were answered using a five-point Likert scale. Some other authors have employed a seven-point Likert scale or have more options answer scale [14]. In this study, the high mean age of the population and level of education made it difficult to have a more complicated answering system. In addition, another inherent problem was the percentage of missing or nonresponding patients and could be attributed to patients with worse health conditions. In this study, 118 patients completed the study out of 200 patients, which is considered satisfactory compared to the length of follow-up after procedure. Although the losses of the volunteers could affect the validity of the sample size, the effect is probably minimal, and the results can be generalized for the whole sample, as seen in other studies [17].

In terms of sample size and representativeness, recruited patients were fewer than expected, especially in the frequency of TKR indications. Another limitation of this study could be that it did not measure patients' expectations before the procedure. Attainment of expectations was highly linked with the level of satisfaction. Patients who stated that their prospects had been met after procedure reported better quality of life [27]. Meanwhile, another study emphasized the importance of developing realistic expectations regarding TKR to avoid dissatisfaction with the surgical outcome [31].

## 5. Conclusion

A TKR procedure can provide an overall improvement in patients' quality of life. These improvements can be detected for several months after the procedure. Function and pain are the most critical indicators of improvement in quality of life. After a TKR procedure, psychological demand decreased significantly to a moderate level. The physical functioning and pain after the TKR procedure was improved significantly, as expressed by the total WOMAC scale. Among the studied demographic factors, gender and age are the main predictors of quality of life among TKR patients. However, other factors such as marital status, educational level, BMI, location, qualification, and site of knee procedure were excluded as predictors in this study. Future studies should consider other patients' groups (e.g., patients with partial knee replacement) and control groups. In addition, other factors should be taken into consideration, such as implant type, patient characteristics, previous health status, and a bigger sample size. Moreover, it is advisable to consider other additional interventions that can help improve the procedure's outcome. The planning for health care resources and the perception of mental and physical activity should be considered, especially in elderly female patients. The application of scales (e.g., WOMAC and SF-36) will give us valuable tools for gathering information before decision-making in health care systems.

## Figures and Tables

**Table 1:** Correlation coefficients and Cronbach's alpha for the scales used in this study.

#	Factor	1	2	3	4	5	Cronbach's alpha
1	Total WOMAC scale	—					0.84
2	Pain severity	0.62^∗∗^	—				0.76
3	Joint stiffness	0.31^∗∗^	0.37^∗∗^	—			0.62
4	Physical functioning impairment	0.71^∗∗^	0.30^∗∗^	0.46^∗∗^	—		0.79
5	Psychological demand (SF-36)	0.28^∗∗^	0.21^∗∗^	0.01	0.22^∗∗^	—	0.76

^∗∗^ *P* value: <0.001.

**Table 2:** Patients' demographics (*N*  = 118).

Variables	Descriptive	Frequency (%)
Gender	Male	54 (45.4)
	Female	64 (53.8)

Marital status	Single	0
	Married	103 (87.3)
	Divorced/widow	15 (12.7)

Age (years)	M ± SD	66.06 ± 6.83

Body mass index	Weight/(height^2^)	26.17 ± 3.32

Location	Middle	87 (73.7)
	South	23 (19.5)
	North	8 (6.8)

Educational level	High school or less	66 (55.9)
	Diploma degree	28 (23.7)
	Bachelor's degree	24 (20.3)
	Postgraduate degree	0

Site of knee procedure	Right knee	56 (47.5)
	Left knee	49 (41.5)
	Both knees	13 (11)

Previous TKR procedure	No	101 (85.6)
	Once	14 (11.9)
	More than once	3 (2.5)

Comorbidities	Not applicable	13 (11.1)
	Diabetes mellitus	17 (14.4)
	Hypertension	19 (8.5)
	Diabetes mellitus + hypertension	59 (50)
	Arthritis	10 (8.5)

M ± SD: mean ± standard deviation.

**Table 3:** The quality-of-life differences among patients before and after TKR (*N*  = 118).

Variables		Before TKR, M ± SD	Status level	After TKR, M ± SD	Status level	*t*-test	*p* value
Psychological demand (SF-36)		3.88 ± 0.37	Severe	2.36 ± 0.21	Moderate	19.13	<0.001^∗∗^
Quality of life (WOMAC scale)	Pain severity	3.95 ± 0.46	Severe	1.86 ± 0.31	Mild	13.45	<0.001^∗∗^
	Joint stiffness	3.91 ± 0.67	Severe	1.79 ± 0.50	Mild	12.43	<0.001^∗∗^
	Physical functioning impairment	4.03 ± 0.30	Severe	1.96 ± 0.26	Mild	14.69	<0.001^∗∗^
	Total WOMAC scale	4.00 ± 0.31	Severe	1.92 ± 0.21	Mild	14.36	<0.001^∗∗^

M ± SD: mean ± standard deviation; TKR: total knee replacement. ^∗∗^*p* value: <0.001.

**Table 4:** Participants' demographics based on groups.

Variables	Descriptive	Group 1, *n* = 38, frequency (%)	Group 2, *n* = 48, frequency (%)	Group 3, *n* = 32, frequency (%)
Gender	Male	25 (21.2)	18 (15.3)	11 (9.3)
	Female	13 (11)	30 (25.4)	21 (17.8)

Marital status	Single	0	0	0
	Married	34 (28.8)	42 (35.6)	27 (22.9)
	Divorced/widow	4 (3.4)	6 (5.1)	5 (4.2)

Age (Years)	M ± SD	66.76 ± 7.05	66.04 ± 6.51	66.25 ± 7.14

Body Mass index	Weight/(height^2^)	25.71 ± 3.34	26.37 ± 3.40	26.41 ± 3.23

Location	Middle	30 (25.4)	37 (31.4)	20 (16.9)
	South	7 (5.9)	9 (7.6)	7 (5.9)
	North	1 (0.8)	2 (1.7)	5 (4.2)

Educational level	High school or less	21 (17.8)	25 (21.2)	20 (16.9)
	Diploma degree	9 (7.6)	13 (11)	6 (5.1)
	Bachelor's degree	8 (6.8)	10 (8.5)	6 (5.1)
	Postgraduate degree	0	0	0

Site of knee procedure	Right knee	16 (13.6)	27 (22.9)	13 (11)
	Left knee	18 (15.3)	16 (13.6)	15 (12.7)
	Both knees	4 (3.4)	5 (4.2)	4 (3.4)

Previous TKR procedure	No	31 (26.3)	42 (35.6)	28 (23.7)
	Once	6 (5.1)	5 (4.2)	3 (2.5)
	More than once	1 (0.8)	1 (0.8)	1 (0.8)

Comorbidities	Not applicable	3 (2.5)	9 (7.6)	4 (3.4)
	Diabetes mellitus	5 (4.2)	9 (7.6)	6 (5.1)
	Hypertension	8 (6.8)	9 (7.6)	4 (8.5)
	Diabetes Mellitus + hypertension	21 (17.8)	16 (13.5)	17 (14.4)
	Arthritis	1 (0.8)	5 (4.2)	1 (0.8)

%: percentage of total. M ± SD: mean ± standard deviation.

**Table 5:** The duration differences of quality of life among patients after TKR (*N*  = 118).

Variables		Group 1, M ± SD	Status level	Group 2, M ± SD	Status level	Group 3, M ± SD	Status level
Psychological demand (SF-36)		2.41 ± 0.21	Moderate	2.33 ± 0.20	Moderate	2.32 ± 0.22	Moderate
Quality of life (WOMAC)	Pain severity	1.97 ± 0.30	Mild	1.84 ± 0.30	Mild	1.74 ± 0.28	Mild
	Joint stiffness	1.95 ± 0.50	Mild	1.73 ± 0.53	Mild	1.70 ± 0.42	Mild
	Physical functioning impairment	2.11 ± 0.20	Mild	1.88 ± 0.22	Mild	1.89 ± 0.25	Mild
	Total WOMAC scale	2.06 ± 0.19	Mild	1.86 ± 0.18	Mild	1.85 ± 0.20	Mild

M ± SD: mean ± standard deviation; TKR: total knee replacement; Group 1: the first 3 months after TKR (*n* = 38); Group 2: between 3 and 6 months (*n* = 48); Group 3: between 6 and 12 months (*n* = 32). ^∗∗^*p* value: <0.001.

**Table 6:** Results of linear regression analysis (stepwise regression) for quality-of-life predictors (*N*  = 118).

Factor	Model	*R*	*R* ^2^	*R* change	Unstandardized coefficient		*t*-test	Sig.
Quality of life (WOMAC)	1^a^	0.609	0.365	0.371	B	Std. error	46.021	<0.001 ^∗∗^
					2.321	0.050		
					0.257	0.031	8.270	<0.001 ^∗∗^
	2^b^	0.628	0.394	0.023	2.641	0.161	16.393	<0.001 ^∗∗^
					0.263	0.031	8.543	<0.001 ^∗∗^
					0.005	0.002	2.093	0.039 ^*∗*^

^a^Predictors: constant and gender. ^b^Predictors: constant, gender, and age. *p* value significant at ^∗∗^<0.01 and ^*∗*^<0.05.

## Data Availability

The data used to support the findings of this study are available from the corresponding author upon request.
